# Supplementary material for: Identification and phylogenetic analysis of the genus Syringa based on chloroplast genomic DNA barcoding
Source: PLoS One. 2022 Jul 19;17(7):e0271633. doi: 10.1371/journal.pone.0271633 (PMC9295972; doi:10.1371/journal.pone.0271633)
Supplement: S1 Table — (PDF) [file pone.0271633.s001.pdf]

S1 Table. Barcode of selected species of the genus *Syringa* based on the variable regions of *psbA-trnH* and *trnC-petN* markers.

Region	*psbA-trnH*																															
**Position**	**2**	**7**	**9**	**10**	**18**	**25**	**38**	**41**	**48**	**49**	**56**	**61**	**62**	**65**	**76**	**101**	**103**	**109**	**110**	**115**	**117**	**128**	**143**	**158**	**162**	**165**	**166**	**170**	**171**	**172**	**176**	**178**	**182**
S. vulgaris 'Macroflora'	-	-	-	T	T	G	T	G	A	A	A	T	G	C	T	T	G	T	G	C	A	T	C	T	T	G	A	G	A	A	G	A	G
S. vulgaris 'Alba Plena'	-	-	-	T	T	G	T	G	A	A	A	T	G	C	T	T	G	T	G	C	A	T	C	T	T	G	A	G	A	A	G	A	G
S. oblata var. *affinis*	-	-	-	T	T	G	T	G	A	A	A	T	G	C	T	T	G	T	G	C	A	T	C	T	T	C	A	G	A	A	G	A	G
S. oblata	-	-	-	T	T	G	T	G	A	A	A	T	G	C	T	T	G	A	G	C	A	T	C	T	T	C	A	-	-	-	G	A	G
S. oblata 'Ziyun'	-	-	-	T	T	G	T	G	A	A	A	T	G	C	T	T	G	A	G	C	A	T	C	T	T	C	A	-	-	-	G	A	G
S. oblata subsp. *dilatata*	-	-	-	T	T	G	T	G	A	A	A	T	G	C	T	T	G	A	G	C	A	T	C	T	T	C	A	-	-	-	G	A	G
S. × *hyacinthiflora* 'Luo Lan Zi'	-	-	-	T	T	G	T	G	A	A	A	T	G	C	T	T	G	A	G	C	A	T	C	T	T	C	A	-	-	-	G	A	G
S. × *hyacinthiflora* 'Asessippi'	-	-	-	T	T	G	T	G	A	A	A	T	G	C	T	T	G	A	G	C	A	T	C	T	T	C	A	-	-	-	G	A	G
S. × *hyacinthiflora* 'Blanche Sweet'	-	-	-	T	T	G	T	G	A	A	A	T	G	C	T	T	G	A	G	C	A	T	C	T	T	C	A	-	-	-	G	A	G
S. × *hyacinthiflora* 'Mount Bake'	-	-	-	T	T	G	T	G	A	A	A	T	G	C	T	T	G	A	G	C	A	T	C	T	T	C	A	-	-	-	G	A	G
S. × *chinensis*	-	-	-	T	T	G	T	G	G	G	C	C	G	C	T	A	T	A	G	A	A	G	T	T	T	T	C	T	C	A	T	A	G
S. × *chinensis* 'Saugeana'	-	-	-	T	T	G	T	G	G	G	C	C	G	C	T	A	T	A	G	A	A	G	T	T	T	T	C	T	C	A	T	A	G
S. tomentella	-	-	-	C	T	T	G	A	G	G	C	C	G	C	T	A	T	A	C	A	A	G	C	T	T	G	A	T	C	A	T	A	G
S. sweginzowii	-	-	-	T	T	G	G	A	G	G	C	C	G	C	T	A	T	A	G	A	A	G	C	T	T	G	A	T	C	A	T	A	G
S. josikaea	-	-	-	T	T	G	T	G	G	G	C	C	G	C	T	A	T	A	G	A	A	G	C	T	T	G	A	T	C	A	T	A	G
S. 'Zhan Mu Shi'	-	-	-	T	T	G	T	G	G	G	C	C	G	C	T	A	T	A	G	A	A	G	C	T	T	G	A	T	C	A	T	A	G
S. × *prestoniae* 'James Macfarlane'	-	-	-	T	T	G	T	G	G	G	C	C	G	C	T	A	T	A	G	A	A	G	C	T	T	G	A	T	C	A	T	A	G
S. × *prestoniae* 'Minuet'	-	-	-	T	T	G	T	G	G	G	C	C	G	C	T	A	T	A	G	A	A	G	C	T	T	G	A	T	C	A	T	A	G
S. emodi	-	-	-	T	T	G	T	G	G	G	C	C	G	C	T	A	T	A	G	A	A	G	C	T	T	G	A	T	C	A	T	A	G
S. wolfii	-	-	-	T	T	G	T	G	G	G	C	C	G	C	T	A	T	A	G	A	A	G	C	T	T	G	A	T	C	A	T	A	G
S. villosa	-	-	-	T	T	G	T	G	G	G	C	C	G	C	T	A	T	A	G	A	A	G	C	T	T	G	A	T	C	A	T	A	G
S. pubescens subsp. *patula*	G	A	C	T	T	G	T	G	G	G	C	C	G	G	T	A	T	A	G	A	A	T	C	T	-	-	-	-	-	-	-	-	-
S. pubescens subsp. *microphylla*	T	A	A	T	T	G	T	G	G	G	C	C	A	C	G	A	T	A	G	A	T	T	C	T	-	-	-	-	-	-	-	-	-
S. pubescens subsp. *microphylla* 'Superba'	T	C	A	T	T	G	T	G	G	G	C	C	A	C	G	A	T	A	G	A	T	T	C	T	-	-	-	-	-	-	-	-	-
S. microphylla 'Superba'	T	C	-	T	T	G	T	G	G	G	C	C	A	C	G	A	T	A	G	A	T	T	C	T	-	-	-	-	-	-	-	-	-
S. meyeri	T	A	A	T	T	G	T	G	G	G	C	C	A	C	G	A	T	A	G	A	T	T	C	T	-	-	-	-	-	-	-	-	-
S. meyeri 'Palibin'	T	A	A	T	T	G	T	G	G	G	C	C	A	C	G	A	T	A	G	A	T	T	C	T	-	-	-	-	-	-	-	-	-
S. 'Si Ji Lan'	T	A	A	T	T	G	T	G	G	G	C	C	A	C	G	A	T	A	G	A	T	T	C	T	-	-	-	-	-	-	-	-	-
S. reticulata subsp. *pekinensis*	-	-	-	T	C	G	T	G	G	G	-	-	-	-	T	A	T	A	G	A	A	G	C	C	T	G	A	T	G	C	T	G	T
S. 'Jinyuan'	-	-	-	T	C	G	T	G	G	G	-	-	-	-	T	A	T	A	G	A	A	G	C	C	T	G	A	T	G	C	T	G	T
S. reticulata subsp. *amurensis*	-	-	-	T	C	G	T	G	G	G	-	-	-	-	T	A	T	A	G	A	A	G	C	C	T	G	A	T	G	C	T	G	T
S. reticulata	-	-	-	T	T	G	T	G	G	G	C	C	G	C	T	A	T	A	G	A	A	G	C	T	C	G	A	T	C	C	T	A	G
S. 'Xiang Ya Duan'	-	-	-	T	T	G	T	G	G	G	C	C	G	C	T	A	T	A	G	A	A	G	C	T	C	G	A	T	C	C	T	A	G

Region	*psbA-trnH*																													
**Position**	**189**	**231**	**236**	**260**	**263**	**272**	**277**	**283**	**286**	**304**	**319**	**361**	**387**	**392**	**394**	**395**	**401**	**402**	**403**	**404**	**406**	**407**	**412**	**413**	**415**	**424**	**431**	**435**	**436**	**437**	**440**
S. vulgaris 'Macroflora'	A	G	T	G	C	C	A	T	T	C	A	A	T	T	T	T	T	T	C	T	T	C	G	G	C	C	A	T	C	T	T
S. vulgaris 'Alba Plena'	A	G	T	G	C	C	A	T	T	C	A	A	T	T	T	T	T	T	C	T	T	C	G	G	C	C	A	T	C	T	T
S. oblata var. *affinis*	A	G	T	G	C	C	A	G	T	C	A	T	T	G	C	C	A	T	A	G	A	C	A	A	A	C	A	T	C	T	T
S. oblata	A	G	T	G	C	C	A	T	T	C	A	T	T	G	C	C	A	T	A	G	A	C	A	A	A	C	A	T	C	T	T
S. oblata 'Ziyun'	A	G	T	G	C	C	A	T	T	C	A	T	T	G	C	C	A	T	A	G	A	C	A	A	A	C	A	T	C	T	T
S. oblata subsp. *dilatata*	A	G	T	G	C	C	A	T	T	C	A	T	T	G	C	C	A	T	A	G	A	C	A	A	A	C	A	T	C	T	T
S. × *hyacinthiflora* 'Luo Lan Zi'	A	G	T	G	C	C	A	T	T	C	A	T	T	G	C	C	A	T	A	G	A	C	A	A	A	C	A	T	C	T	T
S. × *hyacinthiflora* 'Asessippi'	A	G	T	G	C	C	A	T	T	C	A	T	T	G	C	C	A	T	A	G	A	C	A	A	A	C	A	T	C	T	T
S. × *hyacinthiflora* 'Blanche Sweet'	A	G	T	G	C	C	A	T	T	C	A	T	T	G	C	C	A	T	A	G	A	C	A	A	A	C	A	T	C	T	T
S. × *hyacinthiflora* 'Mount Bake'	A	G	T	G	C	C	A	T	T	C	A	T	T	G	C	C	A	T	A	G	A	C	A	A	A	C	A	T	C	T	T
S. × *chinensis*	A	G	T	C	C	C	G	T	T	T	T	T	T	G	C	C	A	T	A	G	A	C	A	A	A	C	A	T	C	T	T
S. × *chinensis* 'Saugeana'	A	G	T	C	C	C	G	T	T	T	T	T	T	G	C	C	A	T	A	G	A	C	A	A	A	C	A	T	C	T	T
S. tomentella	A	G	T	C	C	C	G	G	T	C	T	T	C	G	C	C	A	T	A	G	A	C	A	A	A	T	A	T	C	T	T
S. sweginzowii	A	A	T	C	C	C	G	G	T	C	T	T	C	G	C	C	A	T	A	G	A	C	A	A	A	T	A	T	C	T	T
S. josikaea	A	G	T	A	A	C	G	G	T	C	T	T	-	-	-	-	-	-	-	-	A	C	A	A	A	T	A	T	C	T	T
S. 'Zhan Mu Shi'	A	G	T	A	A	C	G	G	T	C	T	T	-	-	-	-	-	-	-	-	A	C	A	A	A	T	A	T	C	T	T
S. × *prestoniae* 'James Macfarlane'	A	G	T	A	A	C	G	G	T	C	T	T	-	-	-	-	-	-	-	-	A	C	A	A	A	T	A	T	C	T	T
S. × *prestoniae* 'Minuet'	A	G	T	A	A	C	G	G	T	C	T	T	-	-	-	-	-	-	-	-	A	C	A	A	A	T	A	T	C	T	T
S. emodi	A	G	T	A	C	C	G	G	T	C	T	T	C	G	C	C	A	T	A	G	A	C	A	A	A	T	A	T	C	T	T
S. wolfii	A	G	T	A	C	C	G	G	T	C	T	T	C	G	C	C	A	T	A	G	A	C	A	A	A	T	A	T	C	T	T
S. villosa	A	G	T	A	C	C	G	G	T	C	T	T	C	G	C	C	A	T	A	G	A	C	A	A	A	T	A	T	C	T	T
S. pubescens subsp. *patula*	-	-	-	-	-	-	-	-	-	-	-	T	T	T	T	T	T	T	C	T	G	A	G	G	C	C	A	-	T	C	T
S. pubescens subsp. *microphylla*	-	-	-	-	-	-	-	-	-	-	-	T	T	T	T	T	T	T	C	T	T	C	G	G	C	C	T	C	T	T	C
S. pubescens subsp. *microphylla* 'Superba'	-	-	-	-	-	-	-	-	-	-	-	T	T	T	T	T	T	T	C	T	T	C	G	G	C	C	T	C	T	T	C
S. microphylla 'Superba'	-	-	-	-	-	-	-	-	-	-	-	T	T	T	T	T	T	T	C	T	T	C	G	G	C	C	T	C	T	T	C
S. meyeri	-	-	-	-	-	-	-	-	-	-	-	T	T	T	T	T	T	T	C	T	T	C	G	G	C	C	T	C	T	T	C
S. meyeri 'Palibin'	-	-	-	-	-	-	-	-	-	-	-	T	T	T	T	T	T	T	C	T	T	C	G	G	C	C	T	C	T	T	C
S. 'Si Ji Lan'	-	-	-	-	-	-	-	-	-	-	-	T	T	T	T	T	T	T	C	T	T	C	G	G	C	C	T	C	T	T	C
S. reticulata subsp. *pekinensis*	G	G	T	C	C	C	G	-	C	C	T	T	T	G	C	C	A	C	A	G	A	C	A	A	A	C	A	T	C	T	T
S. 'Jinyuan'	G	G	T	C	C	C	G	-	C	C	T	T	T	G	C	C	A	C	A	G	A	C	A	A	A	C	A	T	C	T	T
S. reticulata subsp. *amurensis*	G	G	T	C	C	C	G	-	C	C	T	T	T	G	C	C	A	C	A	G	A	C	A	A	A	C	A	T	C	T	T
S. reticulata	A	G	G	C	C	T	G	-	G	C	T	T	T	G	C	C	A	T	A	G	A	C	A	A	A	C	A	T	T	T	T
S. 'Xiang Ya Duan'	A	G	G	C	C	T	G	-	G	C	T	T	T	G	C	C	A	T	A	G	A	C	A	A	A	C	A	T	T	T	T

Region	*psbA-trnH*																									
**Position**	**442**	**455**	**459**	**462**	**464**	**467**	**469**	**472**	**474**	**475**	**480**	**487**	**492**	**494**	**496**	**501**	**505**	**507**	**508**	**509**	**512**	**520**	**522**	**533**	**538**	**543**	**545**
S. vulgaris 'Macroflora'	T	A	T	T	T	A	T	A	T	C	C	A	T	A	G	C	A	T	A	A	G	T	A	T	A	-	-
S. vulgaris 'Alba Plena'	T	A	T	T	T	A	T	A	T	C	C	A	A	A	G	C	A	T	A	A	G	T	A	T	A	-	-
S. oblata var. *affinis*	T	T	T	T	T	A	T	G	T	C	C	A	T	G	T	C	G	T	A	G	G	T	G	T	A	A	-
S. oblata	T	T	T	T	T	A	T	A	T	C	C	A	T	A	T	C	A	T	A	G	G	T	A	T	A	A	-
S. oblata 'Ziyun'	T	T	T	T	T	A	T	A	T	C	C	A	T	A	T	C	A	T	A	G	G	T	A	T	A	A	-
S. oblata subsp. *dilatata*	T	T	T	T	T	A	T	A	T	C	C	A	T	G	T	C	G	T	A	G	G	T	G	T	A	C	-
S. × *hyacinthiflora* 'Luo Lan Zi'	T	T	T	T	T	A	T	A	T	C	C	A	T	A	T	C	A	T	A	G	G	T	A	T	A	-	-
S. × *hyacinthiflora* 'Asessippi'	T	T	T	T	T	A	T	A	T	C	C	A	T	A	T	C	A	T	A	G	G	T	G	T	A	-	-
S. × *hyacinthiflora* 'Blanche Sweet'	T	T	T	T	T	A	T	A	T	C	C	A	T	A	T	C	A	T	A	G	G	T	G	T	A	-	-
S. × *hyacinthiflora* 'Mount Bake'	T	T	T	T	T	A	T	A	T	C	C	A	T	A	T	C	A	T	A	G	G	T	G	T	A	-	-
S. × *chinensis*	T	A	C	A	A	A	A	A	C	G	T	T	A	A	G	T	A	A	A	A	C	A	A	G	T	-	-
S. × *chinensis* 'Saugeana'	T	A	C	A	A	C	A	A	C	C	C	T	A	A	G	C	A	A	A	A	G	T	A	T	A	-	-
S. tomentella	T	T	T	T	T	A	T	A	T	C	C	A	T	A	G	C	T	T	C	A	G	T	A	T	A	-	-
S. sweginzowii	T	T	T	T	T	A	T	A	T	C	C	A	T	A	G	C	T	T	C	G	G	T	A	T	A	-	-
S. josikaea	T	T	T	T	T	A	T	A	T	C	C	A	T	A	G	C	T	T	C	G	G	T	A	T	A	G	-
S. 'Zhan Mu Shi'	T	T	T	T	T	A	T	A	T	C	C	A	T	A	G	C	T	T	C	G	G	T	A	T	A	A	-
S. × *prestoniae* 'James Macfarlane'	T	T	T	T	T	A	T	A	T	C	C	A	T	A	G	C	T	T	C	G	G	T	A	T	A	G	-
S. × *prestoniae* 'Minuet'	T	T	T	T	T	A	T	A	T	C	C	A	T	A	G	C	T	T	C	G	G	T	A	T	A	G	-
S. emodi	T	T	T	T	T	A	T	A	T	C	C	A	T	A	T	C	T	T	C	G	G	T	A	T	A	-	-
S. wolfii	T	T	T	T	T	A	T	A	T	C	C	A	T	A	T	C	T	T	C	G	G	T	G	T	A	-	-
S. villosa	T	T	T	T	T	A	T	A	T	C	C	A	T	A	T	C	T	T	C	G	G	T	G	T	A	C	-
S. pubescens subsp. *patula*	C	T	T	T	T	A	T	G	T	C	C	A	T	G	T	C	T	T	C	G	G	T	G	T	A	G	A
S. pubescens subsp. *microphylla*	T	T	T	T	T	A	T	A	T	C	C	A	T	A	G	C	T	T	C	G	G	T	A	T	A	G	G
S. pubescens subsp. *microphylla* 'Superba'	T	T	T	T	T	A	T	A	T	C	C	A	T	A	T	C	T	T	C	G	G	T	A	T	A	G	G
S. microphylla 'Superba'	T	T	T	T	T	A	T	A	T	C	C	A	T	A	T	C	T	T	C	G	G	T	A	T	A	G	T
S. meyeri	T	T	T	T	T	A	T	A	T	C	C	A	T	A	T	C	T	T	C	G	G	T	G	T	A	G	-
S. meyeri 'Palibin'	T	T	T	T	T	A	T	A	T	C	C	A	T	A	T	C	T	T	C	G	G	T	G	T	A	G	-
S. 'Si Ji Lan'	T	T	T	T	T	A	T	A	T	C	C	A	T	A	T	C	T	T	C	G	G	T	G	T	A	G	T
S. reticulata subsp. *pekinensis*	T	T	T	T	T	A	T	G	T	C	C	A	T	G	T	C	T	T	C	G	G	T	G	T	A	G	-
S. 'Jinyuan'	T	T	T	T	T	A	T	G	T	C	C	A	T	G	T	C	T	T	C	G	G	T	G	T	A	G	-
S. reticulata subsp. *amurensis*	T	T	T	T	T	A	T	G	T	C	C	A	T	G	T	C	T	T	C	G	G	T	G	T	A	G	-
S. reticulata	T	T	T	T	T	A	T	A	T	C	C	A	T	A	T	C	T	T	C	G	G	T	G	T	A	G	-
S. 'Xiang Ya Duan'	T	T	T	T	T	A	T	A	T	C	C	A	T	A	T	C	T	T	C	G	G	T	G	T	A	-	-

Region	*trnC-petN*																				
**Position**	**43**	**74**	**140**	**171**	**182**	**236**	**244**	**289**	**330**	**357**	**374**	**381**	**397**	**401**	**403**	**404**	**495**	**497**	**498**	**499**	**503**	**505**
S. vulgaris 'Macroflora'	T	T	T	T	C	A	T	G	G	A	G	A	G	T	T	T	A	G	G	G	G	A
S. vulgaris 'Alba Plena'	T	T	T	T	C	A	T	G	G	A	G	A	G	T	T	T	A	G	G	G	G	A
S. oblata var. *affinis*	T	T	T	T	C	A	T	G	G	A	G	A	G	T	T	G	A	G	G	G	G	A
S. oblata	T	T	T	T	C	A	T	G	G	A	G	A	G	T	T	T	A	G	G	G	G	A
S. oblata 'Ziyun'	T	T	T	T	C	A	T	G	G	A	G	A	G	T	T	T	A	G	G	G	G	A
S. oblata subsp. *dilatata*	T	T	T	T	C	A	T	G	G	A	G	A	G	T	T	T	A	G	G	G	G	A
S. × *hyacinthiflora* 'Luo Lan Zi'	T	T	T	T	C	A	T	G	G	A	G	A	G	T	T	T	A	G	G	G	G	A
S. × *hyacinthiflora* 'Asessippi'	T	T	T	T	C	A	T	G	G	A	G	A	G	T	T	T	A	G	G	G	G	A
S. × *hyacinthiflora* 'Blanche Sweet'	T	T	T	T	C	A	T	G	G	A	G	A	G	T	T	T	A	G	G	G	G	A
S. × *hyacinthiflora* 'Mount Bake'	T	T	T	T	C	A	T	G	G	A	G	A	G	T	T	T	A	G	G	G	G	A
S. × *chinensis*	T	T	C	T	C	G	T	G	G	A	G	A	A	A	T	T	A	G	G	G	G	A
S. × *chinensis* 'Saugeana'	T	T	C	T	C	G	T	G	G	A	G	A	A	A	T	T	A	G	G	G	G	A
S. tomentella	T	T	C	T	T	G	T	G	C	A	G	A	A	A	N	G	A	G	G	G	G	A
S. sweginzowii	T	T	C	T	T	G	T	G	C	A	G	A	A	A	T	G	A	G	G	G	G	A
S. josikaea	T	T	C	T	T	G	T	G	C	A	T	A	A	A	T	G	A	G	G	G	G	A
S. 'Zhan Mu Shi'	T	T	C	T	T	G	T	G	C	A	T	A	A	A	T	G	A	G	G	G	G	A
S. × *prestoniae* 'James Macfarlane'	T	T	C	T	T	G	T	G	C	A	T	A	A	A	T	G	A	G	G	G	G	A
S. × *prestoniae* 'Minuet'	T	T	C	T	T	G	T	G	C	A	T	A	A	A	T	G	A	G	G	G	G	A
S. emodi	T	T	C	T	T	G	T	G	C	A	G	A	A	A	T	G	A	G	G	G	G	A
S. wolfii	T	T	C	T	T	G	T	G	C	A	G	A	A	A	T	G	A	G	G	G	G	A
S. villosa	T	T	C	T	T	G	T	G	C	A	G	A	A	A	T	G	A	G	G	G	G	A
S. pubescens subsp. *patula*	C	T	C	-	T	G	A	G	C	A	G	C	A	A	G	G	A	G	G	G	G	A
S. pubescens subsp. *microphylla*	C	T	C	-	T	G	T	G	C	A	G	C	A	A	G	G	A	G	G	G	G	A
S. pubescens subsp. *microphylla* 'Superba'	C	T	C	-	T	G	T	G	C	A	G	C	A	A	G	G	A	G	G	G	G	A
S. microphylla 'Superba'	C	T	C	-	T	G	T	G	C	A	G	C	A	A	G	G	A	G	G	G	G	A
S. meyeri	C	T	C	-	T	G	T	G	C	A	G	C	A	A	G	G	A	G	G	G	G	A
S. meyeri 'Palibin'	C	T	C	-	T	G	T	G	C	A	G	C	A	A	G	G	A	G	G	G	G	A
S. 'Si Ji Lan'	C	T	C	-	T	G	T	G	C	A	G	C	A	A	G	G	A	G	G	G	G	A
S. reticulata subsp. *pekinensis*	T	T	C	C	T	G	T	A	C	A	T	A	A	G	T	T	C	C	T	A	A	T
S. 'Jinyuan'	T	T	C	C	T	G	T	A	C	A	T	A	A	G	T	T	C	C	T	A	A	T
S. reticulata subsp. *amurensis*	T	T	C	C	T	G	T	A	C	A	T	A	A	G	T	T	C	C	T	A	A	T
S. reticulata	T	C	C	T	T	G	T	A	C	C	G	C	A	G	T	T	C	C	T	A	A	T
S. 'Xiang Ya Duan'	T	C	C	T	T	G	T	A	C	C	G	C	A	G	T	T	C	C	T	A	A	T

Region	*trnC-petN*																					
**Position**	**507**	**511**	**512**	**513**	**514**	**515**	**524**	**535**	**554**	**570**	**590**	**616**	**627**	**628**	**645**	**653**	**756**	**764**	**774**	**802**	**825**	**826**	**827**
S. vulgaris 'Macroflora'	T	T	A	G	T	G	C	C	G	A	T	A	C	A	A	A	T	G	C	A	G	G	A
S. vulgaris 'Alba Plena'	T	T	A	G	T	G	C	C	G	A	T	A	C	A	A	A	T	G	C	A	T	G	G
S. oblata affinis	T	T	A	G	T	G	C	C	T	A	T	A	C	A	A	A	T	G	C	A	G	G	A
S. oblata	T	T	A	G	T	G	C	C	G	A	T	A	C	A	A	A	T	G	C	A	G	G	T
S. oblata 'Ziyun'	T	T	A	G	T	G	C	C	G	A	T	A	C	A	A	A	T	G	C	A	G	G	A
S. oblata subsp. *dilatata*	T	T	A	G	T	G	C	C	G	A	T	A	C	A	A	C	T	G	C	A	G	G	T
S. × *hyacinthiflora* 'Luo Lan Zi'	T	T	A	G	T	G	C	C	G	A	T	A	C	A	A	A	T	G	C	A	T	G	G
S. × *hyacinthiflora* 'Asessippi'	T	T	A	G	T	G	C	C	G	A	T	A	C	A	A	A	T	G	C	A	T	G	G
S. × *hyacinthiflora* 'Blanche Sweet'	T	T	A	G	T	G	C	C	G	A	T	A	C	A	A	A	T	G	C	A	T	G	G
S. × *hyacinthiflora* 'Mount Bake'	T	T	A	G	T	G	C	C	G	A	T	A	C	A	A	A	T	G	C	A	T	G	G
S. × *chinensis*	T	T	A	G	T	G	C	C	G	A	T	A	A	G	A	A	T	T	A	A	G	T	G
S. × *chinensis* 'Saugeana'	T	T	A	G	T	G	C	C	G	A	T	A	A	G	A	A	T	T	A	A	T	G	G
S. tomentella	T	T	A	G	C	G	C	C	G	N	T	A	C	G	A	A	T	T	A	A	T	G	G
S. sweginzowii	T	T	A	G	C	G	C	C	G	A	T	A	C	G	A	A	T	T	A	A	T	G	G
S. josikaea	T	T	A	G	C	G	C	C	G	A	T	A	C	G	A	A	T	T	A	A	T	G	G
S. 'Zhan Mu Shi'	T	T	A	G	C	G	C	C	G	A	T	A	C	G	A	A	T	T	A	A	T	G	G
S. × *prestoniae* 'James Macfarlane'	T	T	A	G	C	G	C	C	G	A	T	A	C	G	A	A	T	T	A	A	T	G	G
S. × *prestoniae* 'Minuet'	T	T	A	G	C	G	C	C	G	A	T	A	C	G	A	A	T	T	A	A	T	G	G
S. emodi	T	T	A	G	C	G	C	C	G	A	T	A	C	G	A	A	T	T	A	A	T	G	G
S. wolfii	T	T	A	G	C	G	C	C	G	A	T	A	C	G	A	A	T	T	A	A	T	G	G
S. villosa	T	T	A	G	C	G	C	C	G	A	T	A	C	G	A	A	T	T	A	A	T	G	G
S. pubescens subsp. *patula*	T	T	A	G	T	G	C	C	G	C	T	A	C	G	A	A	T	T	A	A	T	G	G
S. pubescens subsp. *microphylla*	T	T	A	G	T	G	T	C	G	C	T	A	C	G	A	A	T	T	A	A	T	G	G
S. pubescens subsp. *microphylla* 'Superba'	T	T	A	G	T	G	T	C	G	C	T	A	C	G	A	A	T	T	A	A	T	G	G
S. microphylla 'Superba'	T	T	A	G	T	G	T	C	G	C	T	A	C	G	A	A	T	T	A	A	T	G	G
S. meyeri	T	T	A	G	T	G	T	C	G	C	T	A	C	G	A	A	T	T	A	A	T	G	G
S. meyeri 'Palibin'	T	T	A	G	T	G	T	C	G	C	T	A	C	G	A	A	T	T	A	A	T	G	G
S. 'Si Ji Lan'	T	T	A	G	T	G	T	C	G	C	T	A	C	G	A	A	T	T	A	A	T	G	G
S. reticulata subsp. *pekinensis*	C	C	C	C	C	T	C	T	G	A	T	A	C	G	C	A	T	T	A	G	G	G	A
S. 'Jinyuan'	C	C	C	C	C	T	C	T	G	A	T	A	C	G	C	A	T	T	A	G	T	G	G
S. reticulata subsp. *amurensis*	C	C	C	C	C	T	C	T	G	A	T	A	C	G	C	A	T	T	A	G	G	G	G
S. reticulata	C	C	C	C	C	T	C	T	G	A	G	C	C	G	C	A	A	-	A	G	G	G	C
S. 'Xiang Ya Duan'	C	C	C	C	C	T	C	T	G	A	G	C	C	G	C	A	A	-	A	G	G	G	-
