# Supplementary figures and images for: Identification and phylogenetic analysis of the genus Syringa based on chloroplast genomic DNA barcoding
Source: PLoS One. 2022 Jul 19;17(7):e0271633. doi: 10.1371/journal.pone.0271633 (PMC9295972; doi:10.1371/journal.pone.0271633)

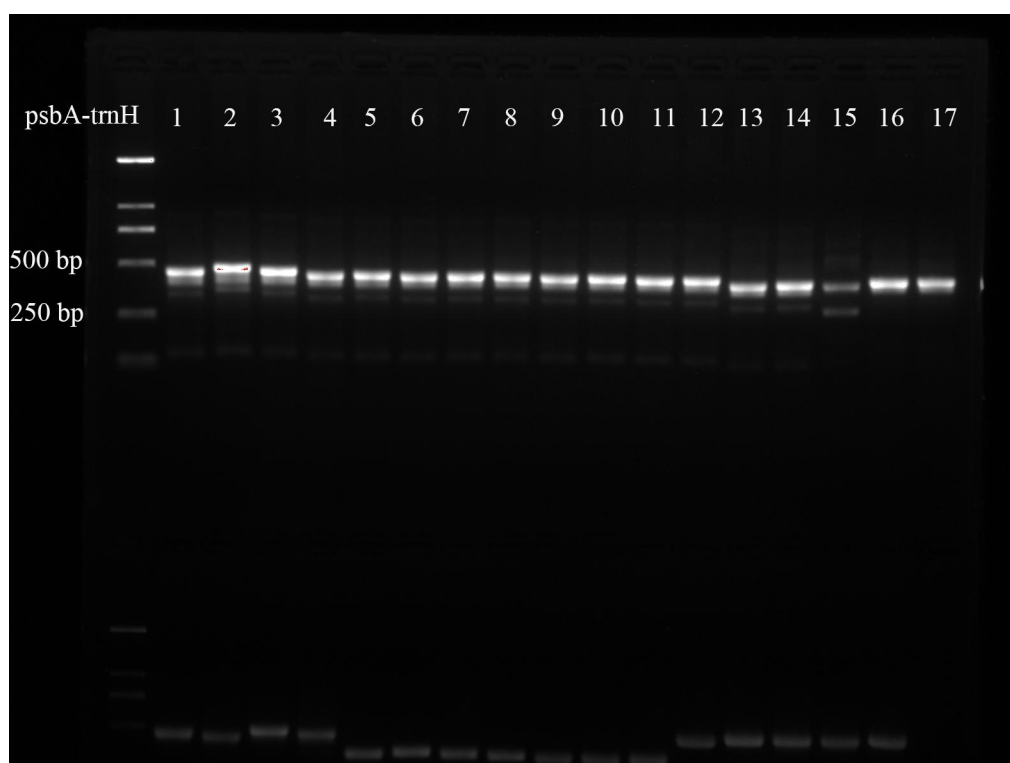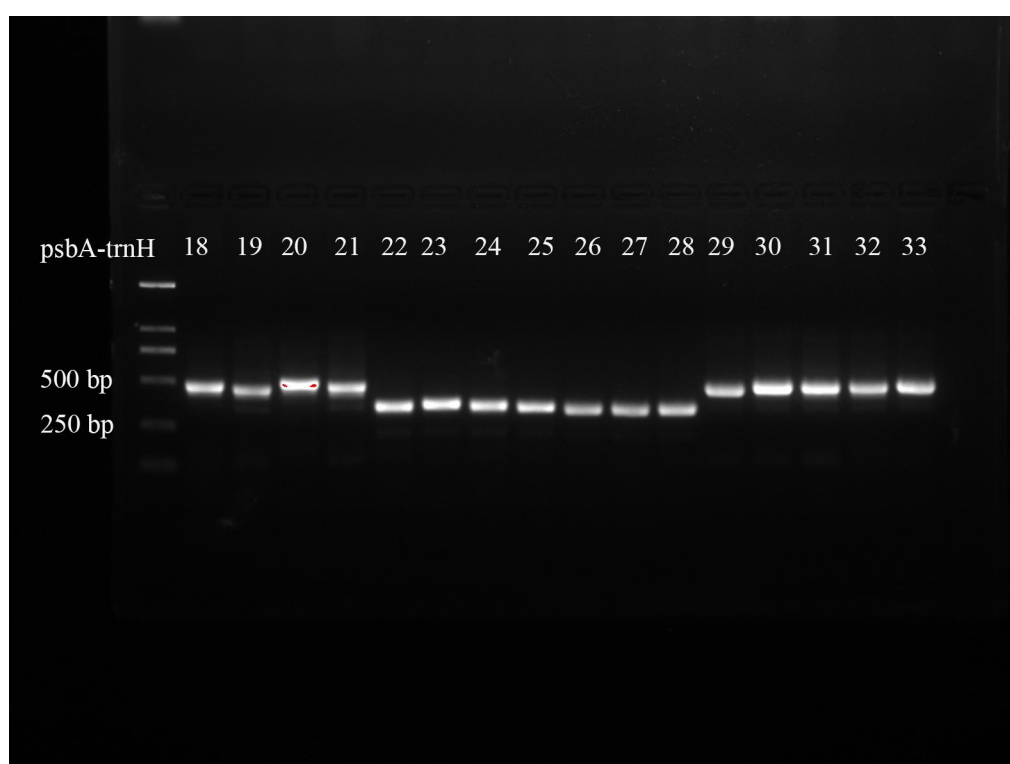

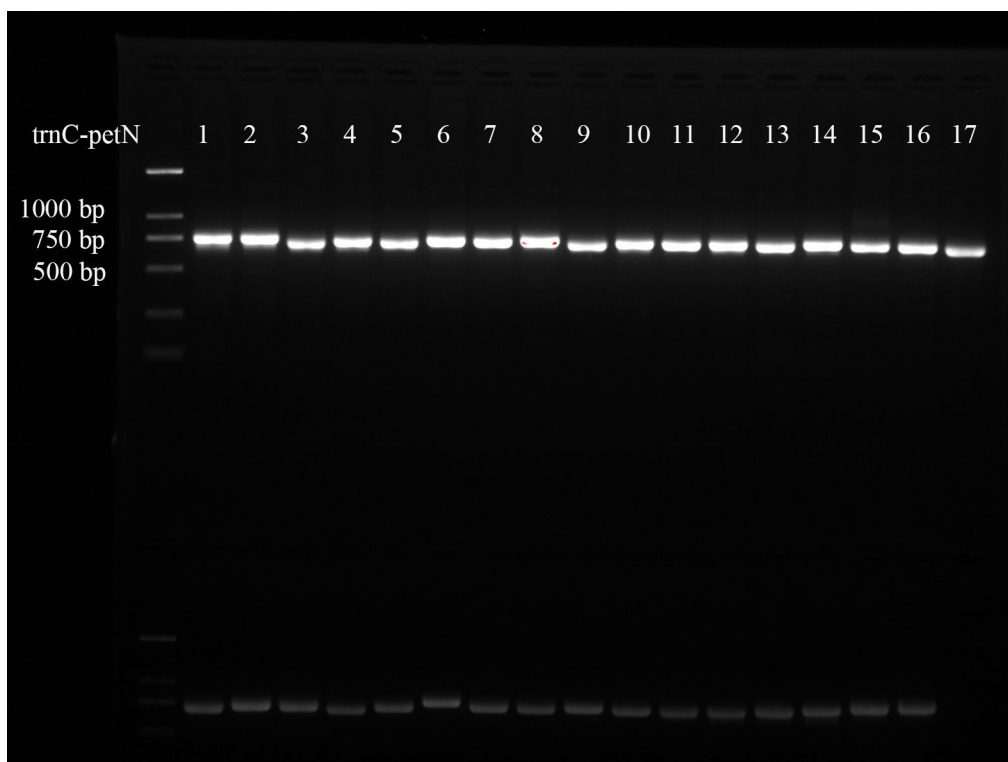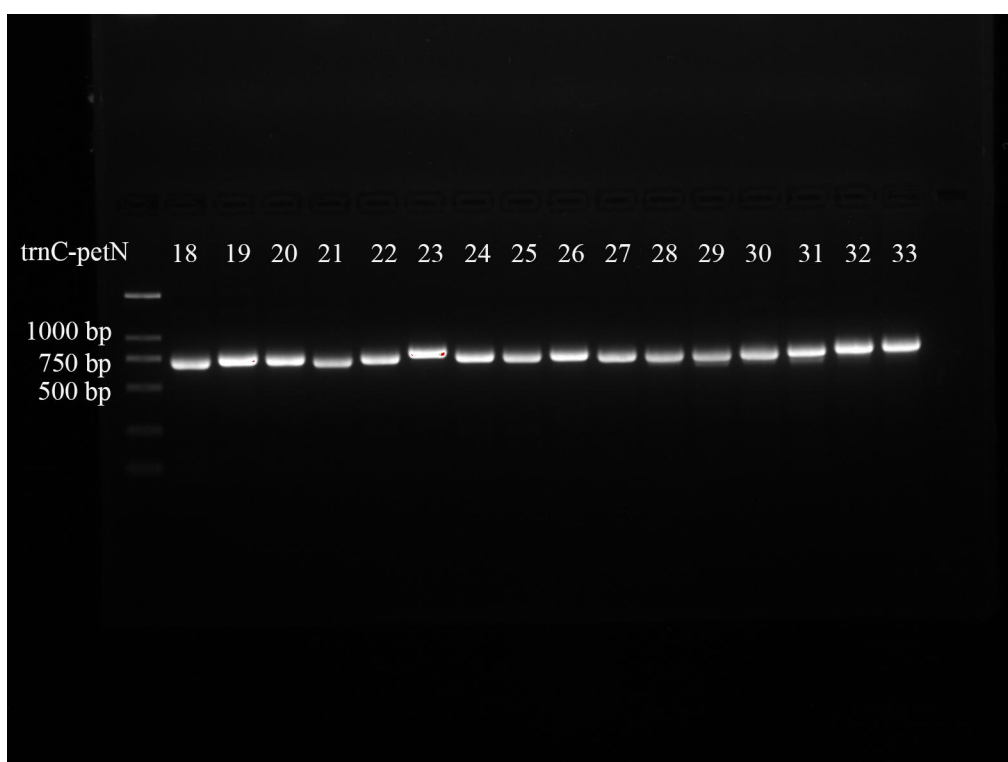

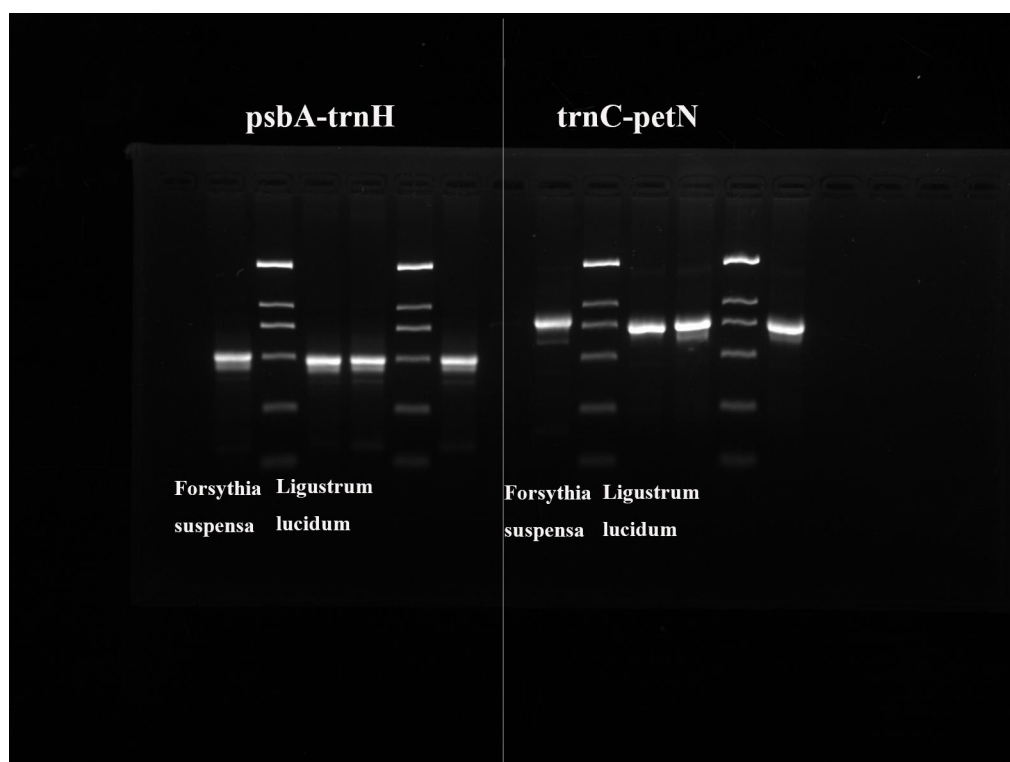

Supplement: S1 Raw images — (PDF) [file pone.0271633.s002.pdf]
