# Supplementary material for: Identification and phylogenetic analysis of the genus Syringa based on chloroplast genomic DNA barcoding
Source: PLoS One. 2022 Jul 19;17(7):e0271633. doi: 10.1371/journal.pone.0271633 (PMC9295972; doi:10.1371/journal.pone.0271633)
Supplement: Attachment — Submitted filename: Response to Reviewers.docx [file pone.0271633.s004.docx]

**Response to Reviewers**

Dear Dr. Branislav T. Šiler:

Thank you very much for giving us an opportunity to revise our manuscript. We appreciate the editor and reviewers very much for their constructive comments and suggestions on our manuscript entitled “Identification and phylogenetic analysis of the genus *Syringa* based on chloroplast genomic DNA barcoding” (ID: PONE-D-22-01886R1).

We have studied reviewers’ comments carefully. According to the reviewers’ detailed suggestions, we have made a careful revision on the original manuscript. All revised portions are marked in red in the “Revised Manuscript with Track Changes” which we would like to submit for your kind consideration.

Kind regards.

Ruihong Yao

E-mail: 337514209@qq.com

Corresponding author : Baosheng Shi

E-mail address: baoshengshi@hebau.edu.cn

Dear Dr. Branislav T. Šiler and reviewers:

Thank you for your letter and the reviewers’ comments on our manuscript entitled “Identification and phylogenetic analysis of the genus *Syringa* based on chloroplast genomic DNA barcoding” (ID: PONE-D-22-01886R1). Those comments are very helpful for revising and improving our paper, as well as the important guiding significance to other research. We have studied the comments carefully and made corrections which we hope meet with approval. The main corrections are in the manuscript and the responds to the reviewers’ comments are as follows (the replies are highlighted in blue ).

Replies to the Editor Comments:

The authors have significantly improved the manuscript. Indeed, the term “genus” was placed in front of “Syringa” throughout the text but not in the main title. Please rectify this. Moreover, please remove the short title to avoid possible misunderstandings.

Response: I’d like to extend my deep gratitude to you for your suggestions. The words “... of *Syringa* based on...” in the main title have been changed to “... of the genus *Syringa* based on...”; “... of *Syringa*” in **line 109** have been changed to “... of the genus *Syringa*”; The words “... of *Syringa* ...” in **line 194** of the S1 Table title have been changed to “... of the genus *Syringa* ...”, and the title of S1 Table in supporting information has also been corrected accordingly. In addition, we have deleted the short title.

Replies to the Journal:

Please review your reference list to ensure that it is complete and correct. If you have cited papers that have been retracted, please include the rationale for doing so in the manuscript text, or remove these references and replace them with relevant current references. Any changes to the reference list should be mentioned in the rebuttal letter that accompanies your revised manuscript. If you need to cite a retracted article, indicate the article’s retracted status in the References list and also include a citation and full reference for the retraction notice.

Response: Sincerely thanks for your kindly suggestions. We have reviewed the references and there are no retracted papers, and each paper can be searched by hyperlink. In terms of the reference format, we have updated as follows: Reference 14 on **lines 370-373** has no page number and volume number (as shown in Fig 1); Reference 16 “Cinnamomum” on **line 380** is updated to “*Cinnamomum*”; the volume issue of Reference 17 on **line 385** is 12 (10); the page number of Reference 23 on **line 410** is 436; Page numbers pp. 76-84 are supplemented to Reference 24 on **lines 411-412**, in addition, we have updated the English title of book from “Flora of China” to “Flora Reipublicae Popularis Sinicae”, as well as “Flora of China” on **line 98** and **line 302** to “Flora Reipublicae Popularis Sinicae”.

Fig 1

Once again, thank you very much for your constructive comments and suggestions which would help us both in English and in depth to improve the quality of the paper.
